# MicroRNAs as Endocrine Modulators of Breast Cancer

**DOI:** 10.3390/ijms26073449

**Published:** 2025-04-07

**Authors:** Vinitha Richard, Kevin Lee, Michael Joseph Kerin

**Affiliations:** 1 Discipline of Surgery, Lambe Institute for Translational Research, University of Galway, H91 V4AY Galway, Ireland; 2 School of Medicine, University of Galway, H91 V4AY Galway, Ireland; k.lee3@universityofgalway.ie

**Keywords:** microRNAs, breast cancer, canonical biogenesis, hormone receptors, systemic biomarkers, circulatory miRNAs, exosomes

## Abstract

Breast cancer is an aggressive disease of multiple subtypes with varying phenotypic, hormonal, and clinicopathological features, offering enhanced resistance to conventional therapeutic regimens. There is an unmet need for reliable molecular biomarkers capable of detecting the malignant transformation from the early stages of the disease to enhance diagnosis and treatment outcomes. A subset of small non-coding nucleic acid molecules, micro ribonucleic acids (microRNAs/miRNAs), have emerged as promising biomarkers due to their role in gene regulation and cancer pathogenesis. This review discusses, in detail, the different origins and hormone-like regulatory functionalities of miRNAs localized in tumor tissue and in the circulation, as well as their inherent stability and turnover that determines the utility of miRNAs as biomarkers for disease detection, monitoring, prognosis, and therapeutic targets.

## 1. Introduction

Breast cancer (BC) accounts for 32% of highly incident cancers and is also the leading cause of cancer-related deaths in women worldwide [1]. Significant advances in imaging technologies in addition to the identification of molecular signatures pertaining to malignant transformation have improved survival [2]. The complexity and heterogeneity of breast cancer present significant challenges for effective diagnosis, prognosis, and treatment [3]. Breast cancer cells display variable expressions of hormone receptors (HRs), mainly estrogen receptors (ERs), progesterone receptors (PRs), and growth factor proteins such as human epidermal growth factor receptor-2 (HER2), and the malignancy encompasses the hormone receptor-based subtypes luminal A (Lum A), luminal B (Lum B), HER2-positive (HER2+), and triple-negative breast cancer (TNBC), each with distinct molecular characteristics and clinical behaviors [3,4,5]. The growth and spread of cancer cells are highly regulated by the hormones estrogen and progesterone (P4), which exert their effects locally via autocrine/paracrine regulation and also enter the circulation to regulate cell–cell communications and cellular signaling mechanisms in metastatic cells at a distant site [5,6]. Similar hormone-like functionality has been displayed by small endogenous noncoding RNA molecules termed microRNAs (miRNAs/miRs), which are 18–25 nucleotides (nt) long and can regulate a wide range of biological and pathological processes [7]. MiRNAs act as chemical messengers that regulate transcriptional or post-transcriptional cellular gene expressions by suppressing the translation of protein coding genes or cleaving target mRNAs to induce their degradation either endogenously or in the tumor microenvironment (TME) [7,8,9]. MicroRNAs have shown particular promise as biomarkers for the early detection of breast cancer as they are highly stable in blood and other body fluids, making them ideal for non-invasive diagnostic tests [10].

Circulating miRNAs (c-miRNAs) have garnered attention as non-invasive liquid biopsy (LB) biomarkers for early and metastatic breast cancer and as a prognostic factor in predicting treatment outcomes in patients with metastatic breast cancer (MBC) after the first line of therapy [11]. Circulating miRNAs encapsulated in exosomes are also reliable biomarkers for breast cancer [12,13]. The diagnostic and prognostic potential of specific circulating miRNAs highlighted in several studies indicates that circulatory miRNAs can act as predictive biomarkers of metastases, long-term survival, responses to neoadjuvant chemotherapy (NAC), and an upregulated expression of miR-145, which correlates to positive outcomes in early-stage breast cancer [14,15,16]. Circulating microRNAs (c-miRNAs), cell-free miRNAs (cfc-miRs) circulating freely in the bloodstream or entrapped in extracellular vesicles (EV-miRs), have been shown to have potential diagnostic, prognostic, or predictive power, with multiple miRNAs significantly elevated in breast cancer patients compared to healthy individuals [17,18].

Considered to be the smallest hormones acting in an autocrine, paracrine, and endocrine manner, microRNAs can also be under the regulation of hormones and act as regulators of both hormones and hormone receptors (HRs) [5,19]. The number of human miRNA candidates identified through high-throughput small RNA sequencing studies now stretches to 2300 true human mature miRNAs, 1115 of which are currently annotated in miRBase V22 [20]. Multiple studies have highlighted a shift from the miRNA-mediated target messenger ribonucleic acid (mRNA) decay or post-transcriptional repression paradigm to include transcriptional activation, epigenetic regulation, and translational upregulation owing to their presence in the powerhouse of a cell, the “mitochondria”, and in the nucleus [21,22]. A subset of miRs localized in the nucleus was found to unconventionally activate gene transcription via a novel phenomenon known as RNA activation (RNAa), which alters histone modification, increases the enrichment of RNA polymerase II (Pol II) and p300 at the enhancer locus, and targets enhancer RNAs (eRNA), which are cis-acting DNA elements that can increase gene transcription [23,24,25]. The selective activation or repression of gene expression is mainly dependent on the key factors, namely, enzymes [Dicer, Drosha] and proteins [Argonautes (Ago1 and Ago2)] involved in miRNA biogenesis and function.

Questions remain for researchers who are yet to address the effective utilization of miRNAs as liquid biopsy markers for the successful management of breast cancer: (i) Is there a disease-specific correlation between the tissue-specific miRNA expressions and the miRNA profile in circulation? (ii) Are circulatory miRNAs a real-time reflection of the presence of malignant cells in breast tissue? (iii) Does tumor tissue undertake specialized mechanisms to efflux a panel of highly upregulated miRNAs out of the tumor tissue to the microenvironment? (iv) Do these secretory miRNAs have a hormonal influence on the tumor microenvironment? (v) How do circulatory miRNAs direct the transformation of tumor cells and neighboring cellular interactants? (vi) Can a differential gain or loss of intracellular microRNAs, similar to endocrinal hormonal expression, be exploited to revert the malignant transformation or be utilized as a potential targeted theranostic? Answers to these questions mainly rely on the specific biogenesis pathways and original localizations of miRNAs in tissues. Through this review, we intend to garner a comprehensive understanding of the endocrine functionalities of the tissue-localized miRs and circulatory miRs of tumor tissue-origin to enhance diagnostic and prognostic accuracy in breast cancer.

## 2. The Biogenesis, Stability, and Functional Roles of MicroRNAs

The discovery of the first miRNA lin-4 in *Caenorhabditis elegans* (*C. elegans*) by Victor Ambros and Gary Ruvkun laid the foundation for miRNA research [26]. High-throughput sequencing technologies have identified the presence of multiple small RNAs (range of 20–30 nucleotides) that can act as regulators of both genes and expressed transcripts [27]. Based on the regulatory roles in heterochromatin formation, mRNA destabilization, translational control, distinct biogenesis mechanisms, limited size (~20–30 nucleotides (nt), and the type of Argonaute protein they are associated with, small RNAs can be broadly classified to three classes: microRNAs (miRNAs), Piwi-interacting RNAs (piRNAs), and endogenous small interfering RNAs (endo-siRNAs or esiRNAs) [27,28]. The significance of microRNAs is that each miRNA may potentially bind to as many as 200 targets, and more than one-third of human genes are predicted to be direct targets of miRNAs [7,29,30].

MiRNAs are evolutionary conserved, single-stranded RNAs (ssRNAs) of ~22 nt in length and function as guide molecules in post-transcriptional gene regulation by base-pairing with the 3′ untranslated region (UTR) of the target mRNAs [27]. The 5′ end of the mature miRNA sequence, from bases 2 to 8, is often called the “seed sequence,’’ which recognizes the 3′UTR of mRNA targets and is required for canonical target binding specificity [7,30,31,32]. The binding of a miRNA to the target mRNA leads to exonucleolytic or endonucleolytic mRNA decay and translational repression [27]. MiRNAs exist as multiple isoforms (paralogues) with identical sequences at nucleotide positions 2–7 relative to the 5′ end, which also embeds the seed sequence of the miRNA thus enhancing base pairing with mRNA targets [27]. There are subclasses of miRNAs classified as “non-canonical miRNAs” derived from alternative non-canonical biogenesis pathways and only partially meet two operational criteria: the expression of a ~22-nt form and the presence of a hairpin precursor [33,34,35,36]. Most functional miRNA-binding sites are canonical in origin and direct translational repression, whereas some of the identified non-canonical sites do not mediate repression of their bound targets [30,37,38]. The biogenesis of miRNAs is a multistep, rapid, and complex process regulated at multiple levels, and it ranks as one of the fastest expressed among transcripts [7,33,34,35,36,37,38,39].

### 2.1. The Canonical Biogenesis of miRNAs

In a typical canonical miRNA biogenesis pathway, RNA Pol II transcribes ~32-nt long primary miRNA (pri-miRNA) transcripts containing one or more hairpin structures that are an imperfectly base-paired stem and a terminal loop containing either coding (mRNA precursors) or non-coding miRNA-transcripts [27,34]. Most canonical pri-miRNAs contain a 5′-cap and a variable poly-A tail [33], and pri-miRNAs in close vicinity often form clusters, giving rise to miRNA families [40,41]. These pri-miRNA transcripts are cleaved by RNase III-endonucleases in two sequential processing reactions, namely, DROSHA in the nucleus and DICER in the cytoplasm, to generate mature miRNAs of approximately 21 nucleotides with a 5′-phosphorylated and 2′-3′-hydroxylated ends [27,28,29]. DICER is essential for the processing of most miRNAs in the cytoplasm of many organisms and cell types [30]. The cleaved pri-miRs yield mature miRNAs with distinct seed regions that may either promote diverse effects or act in a coordinated manner to accomplish common functions [41]. An example of this is the miR-17~92 family of miRNAs consisting of three paralogous miRNA clusters—miR-17~92, miR-106a~363, and miR-106b~25—cleaved to generate 13 distinct mature miRNAs, reported to play an oncogenic role during tumorigenesis [42]. A strand of the mature miRNA (guide strand) has a relatively lower stability of base-pairing at the 5′-end (“the thermodynamic asymmetry rule”), and it associates with a member of the Argonaute (AGO) family of proteins in the cytoplasm to form the core of the RNA-induced silencing complex (miRgonaute or RISC or miRISC), which then interacts and represses target mRNAs [29,30,43]. The second strand (the passenger strand or miRNA*) is degraded [28,31]. MiRNAs also function as effector complexes, interacting with miRNA containing ribonucleoprotein (microribonucleoprotein or miRNP) [44], or with Argonaute, the most important constituent of all miRNPs, favoring the reversal of miRNA-mediated repression to activate the target gene [45]. Translational regulation by miRNPs oscillates between repression and activation as a function of the cell cycle either directly or through the miRNA-mediated abrogation of the repressive action of miRNPs miR [46]. MiRNA directs the association of Argonaute 2 (AGO2) and miRNP (microribonucleoprotein)-associated proteins FXR1 (fragile X mental retardation-related protein 1) with a conserved ARE (AU-rich element) during translational up-regulation and transforms them into effector molecules that bind the ARE to activate translation [46]. Notably, a single miRNA can act in both upregulation and downregulation of target mRNAs in a contextual manner specific to cell type, cell condition, and present factors and elements.

### 2.2. The Non-Canonical Biogenesis of MiRNAs

Contrary to this canonical biogenesis mechanism, a non-canonical pathway independent of DICER and DROSHA exists wherein subclasses of miRNAs classified as “non-canonical miRNAs” originate from other small non-coding RNAs [34]. The non-canonical pri-miRNAs encoded in the introns of coding genes are termed miRtrons [47], and they undergo the splicing process in the nucleus to form stable hairpins with a shorter stem akin to the canonical pri-miRNAs [30,48]. The hairpin structures undergo lariat debranching by the debranching enzyme 1 (DBR1), subsequently cleaved by the RNase III enzyme DROSHA, which forms a microprocessor complex with RNA-binding protein (RBP), the DiGeorge syndrome chromosome region 8 (DGCR8), thereby leaving the debranched intron to form a hairpin structure that resembles a pre-miRNA [48]. This pre-miRNA is protected from degradation and translocated from the nucleus into the cytoplasm by Exportin-5 (XPO-5) in a RAS-related nuclear protein-guanosine-5′-triphosphatase (Ran-GTPase) dependent manner where it is cleaved by DICER [49,50]. In addition to miRtrons, small non-coding miRNAs are derived from other non-coding RNAs, such as transfer RNAs (tRNAs) or small nucleolar RNA (snoRNA) and also exist as DICER-independent miRNAs [30], requiring the AGO2 slicer activity for miRNA maturation [51].

**Figure 1:**
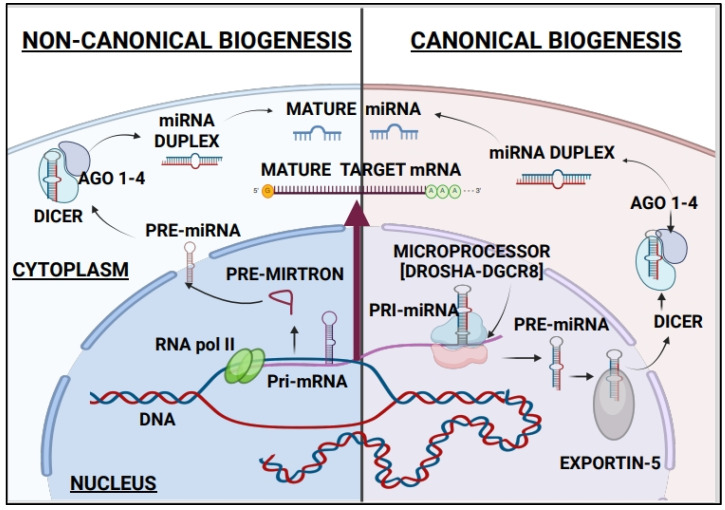
The Canonical and non-canonical pathways for MicroRNA Biogenesis. [Created in BioRender. Richard, V. (2025) https://BioRender.com/bhbgsd7 (accessed on 15 January 2025)].

Conventionally, microRNAs (miRNAs) are short RNA gene regulators originating from primary transcripts cleaved by the nuclear microprocessor complex (DROSHA/DGCR8), resulting in precursor miRNA (pre-miRNA) hairpins exported by XPO-5 and processed by a cytoplasmic Dicer to yield two (5p and 3p) miRNAs. Yet, in another parallel non-canonical microprocessor-independent pathway, 7-methylguanosine (m7G)-capped pre-miRNAs with 5′ ends, coinciding with transcription start sites, and 3′ ends most likely generated through transcription termination that are exported directly to the cytoplasm via exportin-1 [52]. Therefore, it is becoming clear that multiple non-canonical pathways with different processing stages feed pre-miRNAs into the miRNA pathway through Drosha-independent processes and offer additional checkpoints to regulate the master regulator of numerous gene transcripts [Figure 1].

### 2.3. Determinants of Stability and Turnover of MicroRNAs

Approximately 25% of the human genome consists of introns or non-coding gene sequences 4–5 times the size of exons that code for ~25,000 genes [53]. The higher ratio of introns to exons suggests that significant roles in determining species-specific characteristics and complexities are mediated mainly through microRNAs as introns host around 60% of all known microRNAs [54]. The biogenesis of mature miRNAs depends on a few selective genes, namely, *DROSHA*, *DGCR8*, and *DICER1*, to mediate DNA repair and miRNA biogenesis, and *AGO1/2* in guiding miRNA-induced silencing [55]. The microprocessor complex (Drosha/DGCR8) predetermines mature miRNA sequences by generating one end of the mature miRNA, whilst the other pre-existing terminus of the pre-miRNA is cleaved near the terminal loop by Dicer, releasing ~22-nt miRNA duplexes [27,30]. Evidence points towards alterations in miRNA transcripts as an aftermath of DICER1 disruption in cancer [56]. The 22-nt RNA duplex following DICER cleavage is loaded onto an Ago protein to generate the effector complex, RISC, wherein one strand of the RNA duplex remains in Ago as a mature miRNA (the guide strand or miRNA), and the pair strand (the passenger strand or miRNA*) is degraded [27]. Half a million copies of mature miRNAs bound to AGO accumulate per cell and are much more stable than mRNAs [30,39].

Germline pathogenic variants (GPVs) of miRNA biogenesis-related genes, especially *DICER1*, have clinical manifestations (mainly childhood-onset), predisposing the host to hyperplastic and neoplastic conditions predominantly observed in neurodevelopmental diseases (NDD) [55]. Mutational impacts on DICER1 also lead to an increase in -3p miRNAs and a simultaneous decrease in -5p miRNAs, thus influencing the levels of post-transcriptional repression exerted on its mRNA targets [56]. A perfect match in the seed sequence complementarity between the miRNA and the target mRNA in the central region of the miRNA triggers the slicer activity of AGO2 and silences the mRNA targets [57]. Specific interactions of miRNAs with mRNA transcripts containing a near-perfect match sequence result in centered mismatches, followed by the unloading of miRs from AGO, the destabilization of the 3′-end of the miRNA, and eventually, the decay of miRNAs [30,58]. This post-transcriptional regulation of miRNAs is termed target-directed miRNA degradation (TDMD), which also highlights the possibility that miRNAs may accumulate in cells with alterations in AGO1/2 proteins [57,59]. Processing bodies (P-bodies) in the cytoplasm form the main site for executing miRNA-mediated RNA-silencing mechanisms [60]. Additional screening of expressions of *DROSHA*, *DGCR8*, *DICER1*, and *AGO1/2* seemingly has diagnostic and prognostic implications in disorders arising from aberrant expressions of microRNAs. These findings may, at least in part, explain the rapid fluctuations of specific intracellular miRNA concentrations during cell development and cancer.

The rapid and persistent implementation of regulatory functions mediated by microRNAs relies on a stable expression within a cell. From molecular functionalities, miRNAs function like transcription factors that specifically regulate the expression of target genes, whereas biochemically, they resemble relatively stable structural noncoding RNA-protein complexes that can persist for long even after a transcriptional block [35]. Experiments ablating DICER also confirmed that the majority of miRNAs were more stable than mRNAs, with the half-lives of miRNAs expressed both in tissue and serum ranging from 28 to 220 h, 2- to 20-fold longer than that of typical mRNAs (about 10 h) [61]. RNA decay enzymes (RNases) are capable of targeting both the precursors (pri-miRNAs and pre-miRNAs) and mature miRNAs [27]. Argonaute (AGO) proteins are the core component of the silencing complex miRISC that shields its resident miRNA from cleavage by ribonucleases, thereby enhancing the abundance of miRNAs, as well as acting as a silencer [62]. A slow rate of decay prolongs the longevity of mature miRNAs, leading to heightened copy number levels in a single cell, additionally conferring the potential to target multiple mRNAs at the same time [35,63]. The tissue-of-origin and stability of systemic miRNAs in circulation are yet to be explored in detail.

The identification of the extracellular Ago2-miRNA complexes in plasma underlined the mechanism of cellular secretion of the endogenous functional miRNA-induced silencing complex into circulation and has important implications for the development of blood-based biomarker approaches analyzing the circulating miRNAs [64]. Though blood is an RNase-rich environment, miRNAs found in blood are stably expressed and protected from nucleases as extracellular forms encapsulated in membrane-bound vesicles [63]. Numerous systemic miRNAs in biological fluids termed circulating miRNAs remain highly stable in conditions such as low or high pH, repeated freeze and thaw, and long storages at room temperature and reach the receptor cells to execute a distal regulatory role [10,65,66,67]. Cell-free circulating miRNA profiles reflect the homeostatic response and signs of disease progression in an organism and are released from cells in the form of lipid vesicles, microvesicles, exosomes, or apoptotic bodies or as free forms usually bound to ribonucleoprotein complexes (such as Argonaute-2) or high-density lipoproteins (HDL) [65].

**Figure 2:**
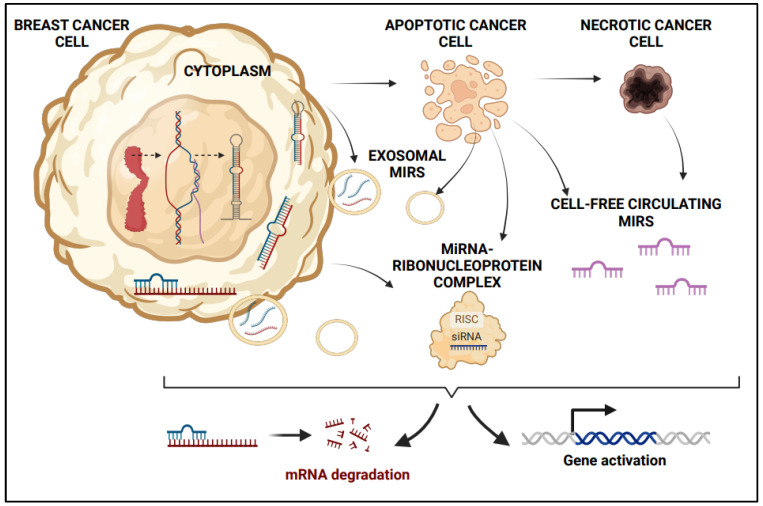
Multiple sources of microRNAs and associated regulatory effects. [Created in BioRender. Richard, V. (2025) https://BioRender.com/lq3wcgn (accessed on 15 January 2025)].

Two main populations of circulating miRNAs identified from plasma are as follows: (i) non-vesicle-associated plasma miRNAs with the Ago2 ribonucleoprotein complex; and (ii) a minority of specific miRNAs associated predominantly with vesicles such as exosomes enable their utilization as blood-based biomarkers for cancer and many diseases alike [64]. Only 10% of cell-free miRNAs released in plasma are through microvesicles, whereas potentially 90% of the miRNAs in the circulation are co-fractionated with ribonucleoprotein complexes [64]. An active process of releasing miRNAs is in the form of exosomes (size < 100 nM) through the course of exocytosis (a process that involves fusion of the multi-vesicular body (MVBs) with the plasma membrane) or through an outward budding from the plasma membrane as microvesicles (100–1000 nM) [65]. An alternative passive process for the release of extracellular miRNAs (EC-miRs) is necrosis or apoptosis (programmed cell death) (PDCD) [65] [Figure 2]. The remarkable fluctuations in the levels of circulating miRNAs reverting to their initial levels after the removal of a tumor enhances the value of cell-free circulating miRNAs (cfc-miRs) as constitutively traceable biomarkers for predicting tumor relapses and for assessing the effectiveness of cancer treatments in breast cancer [15,68,69].

### 2.4. Cell Death and Tumor Turnover Releases MiRNAs in Circulation

Cell death, or cell suicide, is a fundamental process that maintains cellular homeostasis by eradicating mutated or damaged cells in a controlled manner [70]. The three main forms of programmed cell death are apoptosis, autophagy, and programmed necrosis [69]. Apoptosis and necroptosis are cellular processes that act as natural barriers restricting cancer cells from prolonged survival and metastatic dissemination [71]. During necrosis, cells undergo uncontrolled cell death, leading to the release of cellular contents, including miRNAs, into the surrounding environment. This release can result in elevated levels of miRNAs in the bloodstream, reflecting tissue damage or pathological states [71]. MiRNAs can act both as a modulators of cell death mechanisms and as a systemic effectors, thereby enhancing their role in drug-miRNA combination anticancer therapies [72].

Apoptosis, or programmed cell death, also contributes to the secretion of extracellular miRNAs [67]. Apoptotic bodies (Abs), which are membrane-bound vesicles formed during apoptosis may also contain miRNAs, which are released during apoptosis in a controlled process and can play roles in intercellular signaling and immune modulation [67,73]. Phagocytosis of these apoptotic bodies by neighboring cells or immune cells facilitates the transfer of miRNAs and other cellular contents [74]. High rates of apoptosis and necrosis in tumor cells contribute to the release of miRNAs into the bloodstream [75,76]. This release is a natural consequence of the rapid cell turnover characteristic of growing tumors [76]. In addition, circulating tumor cells (CTCs), which are shed from the primary tumor into the bloodstream, can release miRNAs either as they disintegrate or through active secretion [77]. The presence of CTCs and their miRNAs provides critical information about tumor dynamics and metastatic potential, making them valuable for monitoring disease progression and response to therapy [78].

Despite global stability, miRNAs subjected to tight regulation undergo context-based rapid decay dynamics that affect the turnover and cellular homeostasis [35,65]. Substantial dysregulation in miRNA expression levels is a common feature that varies greatly between normal and breast cancerous tissues, as well as between localized and aggressive forms of cancer, depending on the type and stage of the disease [65,66]. The binding of cognate target mRNAs that lays the foundation for miRNA function triggers the stability and decay mechanism of either miRNAs or mRNAs [67]. Cells selectively secrete miRNAs to ensure a moderate regulation of target mRNAs, further influenced by the expression ratio of intracellular miRNAs to target mRNAs (miRNA–mRNA). Specifically, a small miRNA/mRNA ratio tends to retain the miRNAs within the cells, and vice versa favors miRNA exocytosis [67,79]. Irrespective of their stability, miRNAs function as tumor suppressor (tsp) miRs that are inhibitors of tumor growth and act as oncomiRs promoting cancer progression, which makes them potential targets for therapeutic intervention [65,68]. The dysregulated interplay of miRNAs and their target mRNAs promotes the malignant transition through multiple hallmarks of breast cancer.

### 2.5. Imbalance of OncomiRs and Tumor-Suppressor MiRNAs

MicroRNAs act as double-edged swords, promoting oncogenesis or suppressing tumor progression, depending on their target genes [80,81]. A classic example is miR-21, which is frequently upregulated in breast cancer and is known to promote tumor growth and invasion by targeting tumor suppressor genes such as *PTEN* (phosphatase and TENsin homolog) and *PDCD4* (programmed cell death protein 4) [82]. Conversely, miR-34a acts as a tumor suppressor by inhibiting cancer cell proliferation, targeting genes involved in cell cycle regulation, and inducing apoptosis and anti-apoptotic pathways such as *BCL2* (B-cell leukemia/lymphoma 2 protein) and *MYC* (myelocytomatosis oncogene) [83]. MicroRNAs also contribute to the acquisition of chemoresistance by directing regulatory mechanisms that modulate the sensitivity of cancer cells to chemotherapeutic drugs [84]. One such example is miR-200c, part of the miR-200 family, that acts as a tumor suppressor by targeting ZEB1 (zinc finger E-Box binding homeobox 1) and ZEB2 (zinc finger E-Box binding homeobox 2) transcriptional repressors controlling and reducing the expression levels of E-cadherin, which, in turn, plays a crucial role in regulating epithelial–mesenchymal transition (EMT), a process vital for cancer metastasis [85,86]. High expression levels of miR-200c are associated with improved survival rates, suggesting its role as a favorable prognostic marker [87]. In contrast, miR-155 was observed to be frequently overexpressed in triple-negative breast cancer (TNBC), displaying oncogenic function by promoting oncogenesis through various mechanisms, including modulation of immune response and inflammation pathways [88,89]. High expression levels of mir-155 arbitrated the repression of targets SOCS1 (suppressor of cytokine signaling 1) and TP53INP1 (tumor protein 53-induced nuclear protein 1), leading to an enhanced cell proliferation and survival features that correlated with aggressive tumor behaviour and poor patient prognosis [88].

Corroborating findings from previous studies describing an imbalance in the expression of miRNAs endogenously in tissues and exogenously in circulation, which accentuates their potential utility as biomarkers and therapeutic targets, the downregulation of miR-205, miR-375, and miR-182 in breast cancer tissues, coupled with their upregulation in plasma, suggests a dynamic role in tumor progression and metastasis. An active release of these miRs from tumor cells into the circulation, possibly through exosomes, may form part of the adaptation mechanism of cancer cells to systemic environments [89,90,91]. Similar findings have been reported in other studies, where miRNAs such as miR-205 are found to be downregulated in tumors but detectable in exosomal fractions of plasma, indicating their role in intercellular communication and metastasis [92]. The upregulation of miR-34a, and miR-125b in breast cancer tissues, with concurrent downregulation in plasma, aligns with their well-documented roles as tumor suppressors [93]. MiRNA-34a is known for its ability to induce apoptosis and inhibit cell proliferation target genes such as *BCL2* and *MYC* [94]. The loss of this miRNA in circulation could signify its sequestration within the tumor microenvironment, potentially as a response to the cellular stress of tumorigenesis [95]. These findings also echo research from other contemporaries, where the restoration of tumor-suppressive miRNAs like miR-34a and miR-125b was an effective therapeutic strategy in breast cancer [96].

Expression analysis of levels of a second set of miRs, miR-199a and miR-145, exhibited consistent upregulation in tissue but downregulation in plasma, suggesting their robust tumor-suppressive roles as they are involved in inhibiting epithelial–mesenchymal transition (EMT) and metastasis by targeting key transcription factors such as ZEB1 and TWIST1 (twist-related protein 1) [97,98]. The downregulation of these miRNAs in plasma may indicate a loss of their systemic protective effects, a phenomenon observed in other studies where the decreased systemic availability of tumor suppressor miRNAs correlates with increased metastatic potential [72,97,98]. The upregulation of miR-21, miR-1246, and miR-155 in plasma, despite their downregulation in tissues, underscores their role as circulating oncomiRs. These miRNAs are often associated with aggressive tumor phenotypes and poor prognosis, acting by inhibiting tumor suppressor genes such as *PTEN* and *TP53INP1* [99,100]. The elevated levels of these miRNAs in plasma could be reflective of their active release from tumor cells, a mechanism supported by evidence of their enrichment in circulating exosomes [99]. This behavior is consistent with other studies, where miR-21, in particular, is a robust biomarker for breast cancer due to its consistent detection in patient serum [101,102] [Table 1].

The detection of extracellular (EC) or circulating tumor components such as cancer cells, RNA, or circulating tumor DNA (ctDNA) in liquid specimens is collectively termed a liquid biopsy (LB) [111]. Remarkably, the expression levels of miRNAs reflect the various conditions of the body and are promising measurable indicators of BC [126]. Differential expressions of miRs in circulation pre- and post-surgery are predictive of the disease status, prognosis, and treatment impact. Reduced serum levels of miR-155 after surgery and four cycles of chemotherapy in breast cancer patients suggested an elimination of tumor cells, indicating a pathological complete response (pCR), and that miR-155 is an indicator of response to treatment [127]. Despite an escalation in studies identifying miRNAs as promising diagnostic markers and predictors of breast cancer, there is a challenge in establishing a universally recognized panel of accurate and reliable circulating miRNAs as biomarkers owing to their low systemic abundance, variations in sample processing, and our current inability to detect novel miRNAs [65]. The selective release of EC-miRs from viable cancer cells, or cells undergoing apoptosis or necrosis leads to speculation if these small information-imparting molecules may be by-products of dying cells or if they could mediate a warning signal to the TME about an impending cellular dysfunction [66,128]. Similar to hormones in the endocrine system, femtomolar concentrations of miRNAs when released into the bloodstream travel to distant sites to regulate gene expression in target cells throughout the body, indicating their endocrine function [10,128]. The exceedingly low concentration of miRNAs in circulation exerts hormone-like effects on target tissue [128] and are mainly released as exosomes or microvesicles or coupled with Ago2, which enables the effective transfer of EC-miRs between normal and cancer cells, or between cancer cells and the tumor microenvironment (TME) [76].

## 3. Endocrine Regulatory Dynamics of miRNAs

MiRNAs perform a dual mechanism of gene expression regulation, the non-canonical activation of target genes in the nucleus, and the canonical repression of target genes in the cytoplasm [23,129]. Purine or pyrimidine-rich miRNA complementary sequences form triple-helical structures with purine-rich sequences of duplex DNA, favoring triplex formation, chromatin remodeling at enhancer regions, and eventually activating gene transcription [129]. MiRNAs exert their regulatory functions through several modes of action, including autocrine, paracrine, exocrine, endocrine, and circulation [23,66]. In the autocrine mode, miRNAs act on the same cell and modulate gene expression by binding to complementary sequences on target mRNAs, resulting in mRNA degradation or translational repression that maintains cellular homeostasis [130]. Similarly, the paracrine mode involves miRNAs secreted by a cell being taken up by neighboring cells, often mediated by extracellular vesicles (EVs) like exosomes or microvesicles (MVs) or miRNAs bound to RNA-binding proteins [131]. The exocrine mode of EC-miRNA secretion is relevant in glandular tissues wherein endogenous miRs of mammary epithelial cells are secreted into extracellular mammary ducts and transported through ducts to affect distant organs and tissues potentially influencing local tissue environments [130]. Breast cancer cells produce more exosomes per cell than normal mammary epithelial cells, and these could inhibit exosome release from mammary epithelial cells at concentrations of 10 × 10^8^ exosomes per ml, suggesting that tumor cells exert a dominant effect on the regulation of exosomes from normal cells [132]. A dynamic equilibrium exists between exosome release and uptake influenced by the exosome concentration in the extracellular environment that exerts a feedback system regulating the further release of exosomes from normal mammary epithelial cells and breast cancer cells [132].

Analogous to endocrine hormones that enter circulation and regulate the activity of target organs at a distant site, microRNAs, especially the Ago-2 conjugates display hormone-like activities and are important for intercellular communication [5,133]. MiRNAs act as hormones that initiate signaling cascades that change cellular activity in four major ways: (i) miRNAs directly regulate hormone production; (ii) miRNAs directly target genes encoding hormones or enzymes; (iii) miRNAs target hormone receptors and intracellular signaling molecules to alter target cell responses; and (iv) miRNAs are subjected to transcriptional regulation by hormones [133]. Phenotypic traits of TNBC cells transferred successfully by exosomes confirmed an active uptake mechanism of extracellular labelled exosomes [134]. Similarly, miRNAs can also affect hormone receptor (HR) expression and activity [135].

### 3.1. MiRNAs as Regulators of Hormone Receptors and Breast Cancer Subtypes

Studies have reported that miRNAs target estrogen receptor alpha (ERα) receptor proteins and determine the ERα-positive status of breast cancers [135,136]. A reciprocal interaction also co-exists in breast cancer cells whereby miRNAs are under the regulatory control of the ERα transcription factor [135,137]. Although ERα activation modulates expression of multiple miRNAs, the reverse action of the miRNA-mediated regulation of ERα expression and subsequent downstream oncogenic signaling pathways lead to acquired resistance to standard first-line endocrine therapies [138,139] Upregulation of Let-7 and miR-29a in ERα− breast cancer cells collectively target and repress Dicer1, triggering a loss of differentiation and enhanced aggressiveness akin to the TNBC subtype [138,140].

Progesterone receptor (PR) is another nuclear receptor regulated and co-expressed by ERα and the progesterone (P4) hormone, and progesterone receptors (PRs) play significant roles in breast cancer and normal mammary gland development [135]. Experimental evidence proves that miRNAs also control PR expression by binding to a non-coding RNA that spans the PR promoter and not via the conventional mechanism of targeting the 3′UTR of the transcript [141]. Let-7c 142 and members of the miR-520 family [142] were found to positively correlate with PR status but not ERα in breast cancer. A circulatory miR, miR-155, was significantly higher in the sera of women with PR+ breast cancers [143]. A significant upregulated expression of two components of the miRNA biogenesis pathway, Dicer1 and Exportin-5, by P4 suggests that treatments targeting P4 could have a global impact on miRNA expression and vice versa [143].

**Figure 3:**
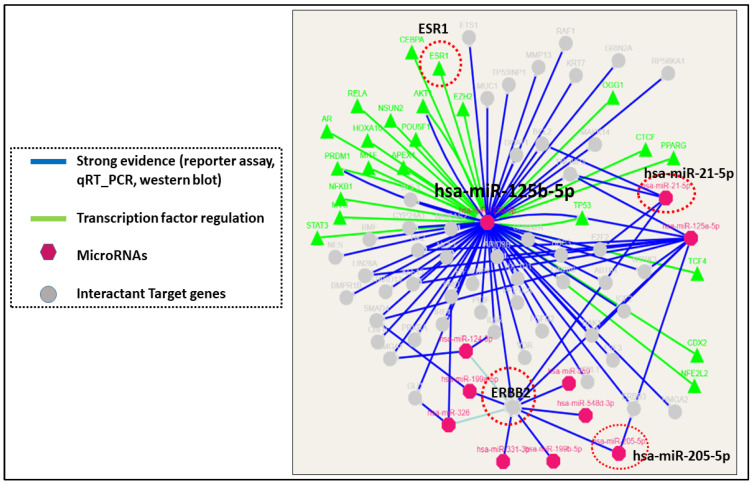
MicroRNAs and target interactions. [Image created using miRTarBase software Release 9.0 beta [https://miRTarBase.cuhk.edu.cn/] accessed on 2 September 2024. [153]. Strong experimental evidence shows that Hsa-miR-125b-5p is one among the multiple miRNAs [hsa-miR-21-5p, hsa-miR-205-5p, and hsa-miR-125a-5p] that targets the *ERBB2* gene in breast cancer and is under the transcriptional regulation of ESR1 transcription factor.

Depicted as a potential breast cancer subtype specific diagnostic biomarker [9], microRNAs can also have a predictive and prognostic significance [144]. In luminal B HER2+ breast cancer, miR-210, miR-4516, miR-718, and miR-125b-5p were specifically associated with chemo-sensitivity [145] and high levels of miR-125b-5p during neoadjuvant chemotherapy [NAC] treatment predicted a poor disease free survival [DFS] [146]. MicroRNA-125b is one of the most down-regulated miRNAs in breast cancer and is able to modulate *ERBB2/3* expression by acting as a double-faced gene expression regulator with tumor suppressor function in solid tumors and an oncogenic role in hematologic malignancies [147] [Figure 3]. Though the luminal A and B tumor subtypes have similar miRNA clusters, there are few dysregulated miRs expressed distinctly from each subtype; for example, miR-1290 is an oncogenic miRNA significantly reduced in Ki67 low luminal A tumors, and its decline correlated with better clinical outcome mainly observed in this breast cancer subtype [148]. In addition, the miR-30 cluster [miR-30c-5p, miR-30b-5p] and the cluster of miR-99a/let-7c/miR-125b forms a miRNA signature for the luminal A subtype [112,149,150,151], whereas the luminal B subtype was enriched for a signature consisting of miR-15b-3p, miR-182-5p, miR-200b-3p, miR-149-5p, miR-193b-3p, and miR-342-3p [152]. The increased expression of tumor suppressor miRNAs predominantly in luminal A breast cancers correlated with the relatively slow growth displayed by luminal A breast cancers in patients.

HER2 positive breast cancer represents a specific breast tumor subtype that typically responds to anti-HER2 therapy [154]. Despite the successful administration of targeted therapy drugs, approximately 50% of HER2+ breast cancer patients fail to benefit from this therapy, and the reason for this poor response was a significant dysregulated expression of miRNAs [miR-23b-3p, miR-195-5p, miR-656-5p, and miR-340-5p] that led to trastuzumab resistance [155]. The intronic miRNA-4728 originating from an excised intron of the HER2 pre-mRNA has two mature forms of isoforms (miR-4728-5p and miR-4728-3p) and has been demonstrated to be functional and significantly upregulated in HER2+ breast cancer patients [154,155]. MiR-4728-5p triggers a positive feedback loop to promote HER2-dependent tumor progression, while the isoform miR-4728-3p downregulated estrogen receptor 1 (ESR1) expression [155,156]. The dual role of miR-4728 promotes cell survival, reduces sensitivity to hormonal therapy by ESR1 down-modulation, and increases response to anti-HER2 therapy by directly targeting ErbB3-binding protein 1 (EBP1) [156,157]. Furthermore, the potential toxicities associated with HER2-targeted drugs, such as cardiotoxicity, skin rash, pain, and insomnia underscore the need for clinical research to identify alternate reliable predictive biomarkers that would help in the early identification of patients likely to respond to therapy, thereby avoiding the overtreatment of patients and lowering of debilitating side effects [157,158,159]. Thus, miRNAs are seemingly versatile clinical tools as therapeutics and reliable biomarkers.

### 3.2. Hormonal Regulation of MiRNAs and Targeted Endocrinal Signalling Pathways

Experimental evidence generated in breast cancer cell lines has proven that hormones, particularly estradiol [E2], can regulate specific miRNAs through both genomic (transcriptional) and non-genomic (membrane-initiated) mechanisms, thus influencing their endocrine-like functions systemically, impacting cell survival, proliferation, and metastasis [160]. In the genomic mechanism, estrogen binds to ERα in the nucleus, activating or repressing transcription of miRNA genes, whereas plasma membrane ERα or G protein-coupled estrogen receptor 1 (GPR30) mediates miRNA regulation in the non-genomic mechanism [160]. E2-ERα mediated indirect regulation of miRNA expression through rapid upregulation of cMYC, while direct suppression of mature miR let-7g conducted by E2 in a mitogen-activated protein kinase (MEK/MAPK)-dependent manner was significantly associated with lymph node metastasis and poor survival in breast cancer patients [161]. The influence of E2 tends to predominantly upregulate the expression of ERα enhancer transcripts, whilst the intergenic non-coding transcripts enriched for miRNAs are predominantly downregulated [160]. Liganded ERα (E2-ERα) suppresses the Drosha-mediated processing of pri-miRNAs to pre-miRNAs, thus executing the suppression of multiple miRNAs and repressing the expression of mature miRs, i.e., miR-125a and miR-145 [162]. A downregulated systemic expression of tumor suppressor miR-145 correlated with poor outcomes in early-stage breast cancer [15]. Conversely, silencing of miR-221/222 that target Erα increased the expression of intrinsic ERα and sensitized the ERα-negative, endocrine therapy-resistant breast cancer cells to tamoxifen-induced apoptosis [163]. Overexpression of an oncogenic isoform of HER2 (HER2Δ16) reduces the expression of miR-15a and miR-16 that targets *BCL-2* and induces tamoxifen resistance luminal A breast cancer subtype [164].

Alternatively, other prominent steroid hormones, such as estrogens, progestins, and androgens, all regulate miRNAs, and the balance of the three receptors contributes to the regulation of breast cancer stem cells (CSCs) [137,140,165,166]. Estrogens bind to ERα and increases the transcription of let-7 and miR-29abc and binds GPR30 to initiate EGFR and Hippo signaling pathways. Progesterone or progestins bind to PR to downregulate GATA3 (GATA3 upregulates expression of miR-29b), miR-141, or miR-29abc and increase CSC populations. Androgens or progestins bind to the androgen receptor (AR) to suppress let-7 microRNA transcription [167]. ER upregulates expression of the miR-29 family; opposing the effects of progesterone as an inhibition of miR-29a or miR-29b alone was sufficient to increase the tumor-initiating ability of CD44+ breast cancer cells [167,168]. The rapid downregulation of miR-141, a tumor suppressor miR, by progesterone potentiated a progesterone-dependent increase in the CD44+ and CK5+ populations, suggesting that suppression of miR-141 helps maintain a more stem-like phenotype that drives the disease [169]. Contradicting the functions of PR, ER further upregulates the expression of the let-7 miRNA family involved in the maintenance of differentiation in normal and cancer cells and downregulates the expression of miR-221/222 that is found in higher levels in CD44+CD24− breast CSCs [164,167,169]. A breast CSC phenotype is determined by the collective effort of three hormones and the miRNAs they regulate, ER upregulates miRNAs that maintain a more differentiated phenotype, while PR, and probably AR, suppress the miRNAs that support breast cancer cell differentiation [167]. The changes in miRNA expression regulated by hormones can have systemic effects, influencing the behavior of breast cancer cells throughout the body and potentially contributing to metastasis and treatment resistance.

## 4. MiRNAs as Inducers and Mediators of Therapeutic Resistance

The presence of miRNAs in circulation, facilitated by various secretion mechanisms, plays a significant role in the development of therapeutic resistance in breast cancer [170]. Tumor cells can use miRNAs as tools for intercellular communication, spreading resistance traits among the tumor population. For instance, exosome-mediated transfer of miRNAs can convey resistance to chemotherapeutic agents by altering the expression of genes involved in drug metabolism, apoptosis, and cell cycle regulation. This exosome-mediated communication contributes to the heterogeneity within the tumor, allowing resistant clones to survive and proliferate despite treatment. Furthermore, the secretion of miRNAs into the circulation may reflect the tumor’s adaptive response to therapeutic pressures, leading to the development of resistance. By modulating the tumor microenvironment and altering the behavior of surrounding cells, circulating miRNAs can promote survival mechanisms that enable the tumor to evade the effects of chemotherapy or targeted therapies. This process underscores the complexity of therapeutic resistance and highlights the potential of targeting miRNA pathways to overcome resistance and improve treatment outcomes. Regulation of tumor exosome release could provide a new therapeutic strategy. Tumor exosomes induce a variety of deleterious responses in the patient, and the inhibition of these responses would be advantageous.

Certain miRNAs downregulate the genes responsible for drug uptake or upregulate the expression of drug efflux pumps, thereby reducing the effectiveness of chemotherapeutic agents [85]. This consecutively limits the intracellular concentration of chemotherapeutic agents, while the upregulation of efflux pumps can enhance the removal of drugs from cancer cells, both of which contribute to resistance. Monitoring these miRNAs in the bloodstream can offer insights into the development of resistance to therapies and help tailor treatment strategies accordingly. Moreover, circulating miRNAs themselves can serve as therapeutic targets. Strategies to inhibit oncogenic miRNAs or restore the function of tumor-suppressive miRNAs provide new avenues for cancer treatment by directly targeting the molecular mechanisms driving the disease [80]. These approaches hold promise for overcoming therapeutic resistance and enhancing the effectiveness of cancer therapies. Among other examples, the transfer of exosomes from stromal cells to breast cancer cells can excite antiviral signaling and alter radiation sensitivity [171]. Adriamycin-resistant breast cancer cells-released exosomes can transmit the resistance capacity to sensitive breast cancer cells by transferring specific miRNAs [172]. The eradication of exosomes expressed by HER2+ breast cancer might prove a useful therapeutic adjuvant [173].

In a role reversal, miRNAs mediating breast cancer can also have therapeutic potential and act as therapeutic targets via the depletion of oncogenic miRNAs by delivering an oligomer (antagomirs) complementary to the target mature miRNAs or the enrichment of tumor suppressive miRNAs [148,173]. Antagomirs are chemically synthesized synthetic double-stranded RNAs that mimic mature endogenous miRNAs, anti-miRNA oligonucleotides (also known as AMOs) that are complementary to a mature miRNA, which they are designed to neutralize [174]. The antagomirs inhibits and degrades target miRs, whereas miRNA mimics the tumor suppressor miRs that are intrinsic to the cell [150,175].

With profound anti-metastatic and anti-proliferative properties, miR-21, miR-10-b, and miR-34a displayed immense preclinical therapeutic potentials [176]. Combination treatment using doxorubicin and miR-34a mimic effectively mitigated cancer cell migration and proliferation in in vitro and in vivo models of TNBC subtype [176]. The Let-7 miRNA family can also act as a tumor suppressor by inhibiting ERα-mediated cellular malignant growth in breast cancer [177]. The miR-200 family represents a prominent cluster of tumor suppressor miRNAs that inhibit epithelial–mesenchymal transition (EMT), migration, invasion, tumor cell adhesion, and metastasis enhancing the possibility to be utilized for targeted therapy [176]. For both strategies to be effective in a clinical setting, the inhibition of oncogenic miRNA activity and the restoration of miRNA activity of tumor suppressor miRNAs need to be highly cell type-specific and ensure minimal toxicity. The wide gap in successful in vitro and in vivo applications of miRNAs in therapy depends on the low stability of exogenously delivered miRNAs that is degraded by nucleases and promptly cleared from the circulatory system [177]. Quantitative estimation of physiologically relevant levels of antagomirs with high tissue specificity and minimal potential off-targets are essential for optimized delivery and uptake by cancer cells [178].

## 5. Limitations of MicroRNAs in Breast Cancer Research

**Figure 4:**
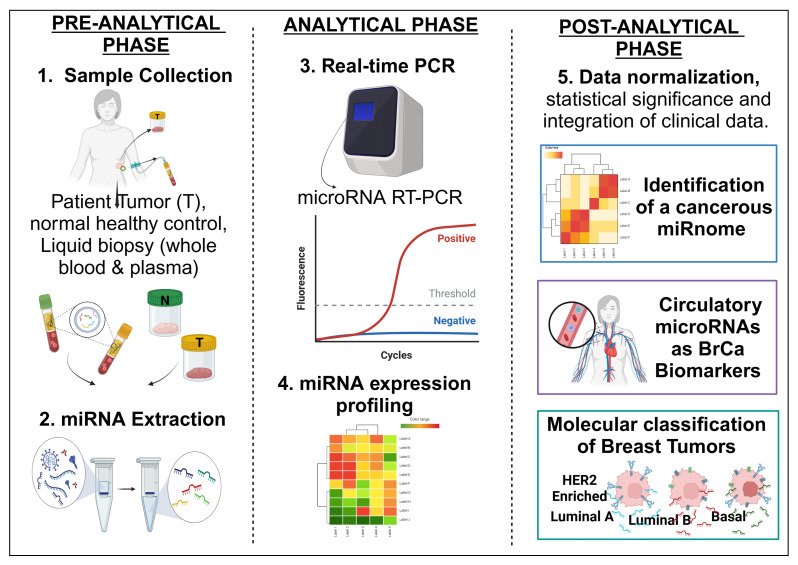
The workflow for miRNA detection. [Created in BioRender. Richard, V. (2025) https://BioRender.com/miutyan (accessed on 15 January 2025)]. The detection of miRNAs from clinical samples includes RNA extraction, miRNA enrichment, reverse transcription, quantitative validation of expression analysis using techniques like microarrays, RT-qPCR, or high-throughput sequencing, followed by statistical data analysis and integration with clinical–pathological features and the differential miRNA expression profile in patients.

Thirty-two years after the landmark discovery of miRNAs by Lee and Ambros [26], clinical trials utilizing microRNAs in diagnostic, therapeutic, or prognostic purpose against breast cancer are still in their infancy stages. Questions remain and pertain to all time points in miRNA research, from the choice of starting material to the methods of sample collection and processing as part of the pre-analytical phase, followed by the analytical phase wherein detection techniques are employed, and finally, the post-analytical steps involved with data analysis, data normalization, and the integration of clinical data [179] Approximately 60 to 70% of errors affecting the accuracy and reliability of molecular biology tests occur at the pre-analytical phase in the laboratory setting itself [180,181]. The impeding factors influencing the final miRNA profile can be categorized to multiple stages: (i) the timing of sample collection; (ii) variations in miRNA concentrations at the time of collection; (iii) specimen type [tissue or blood]; (iv) sample volume; (v) processing of tissues and body fluids post-collection; (vi) procedures for total RNA extraction and the (vii) enrichment step for small miRNAs; (viii) miRNA quantification protocols; and (ix) the final normalization against suitable endo controls in data analysis [181] [Figure 4].

Advancements in the field of miRNA diagnostics have improved tremendously from the gold standard PCR method of basic nucleic acid amplification to highly sensitive techniques such as droplet digital PCR (ddPCR), surface-enhanced Raman spectroscopy (SERS), electrochemiluminescence (ECL), and mass spectrometry (MS) [180], as well as benefitting from the utilization of enhanced high-throughput commercial platforms such as Nanostring’s nCounter ^®^ microRNA assay, as well as next-generation RNA sequencing (RNAseq) [180,181]. Pre-analytical and post-analytical process-induced variability arising from molecular biology experiments requires the establishment of a standardized strategy following on from the first step, i.e., sample collection, to data analysis. The most direct approach to studying the differential expression in cancers would be to screen for miRNAs in the tissue of interest itself [181]. The challenges of obtaining tissues paved the foundation for alternative approaches such as liquid biopsies and blood and plasma screening for the presence of disease-specific miRNAs [182]. Further challenging the steps of accurate detection and quantification, circulating miRNA concentrations are considerably lower than those within cells and tissues [183]. Key innovations in lateral flow assays (LFAs) include signal amplification using isothermal methods, the application of CRISPR (Clustered Regularly Interspaced Short Palindromic Repeats)/Cas systems for the direct targeting of miRNAs, and the incorporation of gold nanoparticles and nanorod nanomaterials to enhance signal intensity, which have emerged as technically simple, affordable, and portable assays or kits ideal for point-of-care testing tools for the rapid and reliable detection of miRNAs [181]. This has enabled the detection of miR-21 levels as low as 20 pico Molar [pM] and let-7a levels as low as 40 pM in a short span of time, i.e., ~ten minutes, thus highlighting the potential of these devices for clinical diagnostics [182,183].

With technological advancements in high-throughput sequencing data, multiple isoforms and novel putative miRs revealed require functional characterization and annotation, thereby enlarging the panel of potential miRNA candidates that needs screening based on the disease context. With more than 10 probable mRNA targets complementary to the seed sequence at the 3′UTR end of a single miRNA, there exists the possibility of a broad range of miRNA–mRNA interactions altering multiple signalling pathways all at once, which includes both transcriptional repression and activation evidenced as clinicopathological transitions. Second, there is limited clarity on the identity of the origins of disease-promoting systemic miRNAs, whether they are released from exosomes, microvesicles, Argo-2 conjugates of viable cells or are they released from dying necrotic or apoptotic cancer cells. Complicating the molecular analysis of miRNAs further is the crucial factor of breast tumor heterogeneity and the presence of various clones with distinct molecular features that questions the level of correlation between tissue-specific and systemic expression of miRNAs. The establishment of an accurate and reliable panel of unanimously agreed robust internal controls for both endogenous tissue-localized miRs and circulating miRs utilized for breast cancer diagnosis still remains a challenge, in addition to other steps from sample collection and processing to data analysis [184,185,186].

The questions raised in the introductory part of this review need to be addressed through ongoing research. Deriving a broad consensus on internationally accepted SOPs within the scientific community for sample processing, testing, and validation supported by open-access data-sharing repositories could lead the way to exploiting the clinical utility of miRNAs as diagnostic markers [186]. MicroRNAs have promising applications as therapeutic inhibitors of tumor cells, many of which are still in the preclinical stage (in vitro and in vivo models), and only a few of them are undergoing clinical trials. In conclusion, miRNA-based therapeutic approaches to overcome BC resistance are very promising and could help to identify tumors with worse prognoses and with different responses to a specific therapy. In addition, miRNA signatures are used to stratify patients and to design personalized approaches for BC treatment. Defining the most relevant miRNAs and standardizing the detection of specific and effective signatures. Moreover, miRNA-based delivery strategies could lead to a relevant change in the management of BC patients and improve diagnosis, prognosis, and overall survival.

## Figures and Tables

**Table 1:** Differential expression of endogenous and systemic miRNAs in breast cancer.

MicroRNA	Expression in Tumor Tissue	Expression in Plasma/Serum	Circulation Source	Reference
miR-205	Downregulated	Upregulated	Serum	[103]
miR-375	Downregulated	Upregulated	Plasma	[91]
miR-182	Downregulated	Upregulated	Plasma	[104]
miR-148b	Downregulated	Upregulated	Plasma	[105]
miR-16	Downregulated	Upregulated	Plasma	[106]
miR-34a	Upregulated	Downregulated	Plasma	[107,108,109]
miR-125b	Upregulated	Downregulated	Plasma	[110,111]
miR-145	Upregulated	Downregulated	Plasma	[111,112,113]
miR-199a	Upregulated	Downregulated	Plasma	[114]
hsa-miR-16	Downregulated	Upregulated	Plasma	[113]
hsa-miR-21	Downregulated	Upregulated	Plasma	[99]
hsa-miR-1246	Downregulated	Upregulated	Plasma	[99]
hsa-miR-155	Downregulated	Upregulated	Plasma	[100]
hsa-miR-215	Downregulated	Upregulated	Plasma	[111,115]
hsa-miR-122-5p	Downregulated	Upregulated	Plasma	[111,115]
hsa-miR-195	Downregulated	Upregulated	Plasma	[111,116,117]
hsa-miR-93	Downregulated	Upregulated	Plasma	[111,118]
hsa-miR-126	Downregulated	Upregulated	Blood	[111,119,120]
hsa-miR-133a-3p	Downregulated	Upregulated	Plasma	[111,119,121]
miR-505-5p	Downregulated	Upregulated	Plasma	[111,119]
miR-125b-5p	Downregulated	Upregulated	Serum	[110,111,119]
has-miR-96	Upregulated	Upregulated	Plasma	[119,122]
hsa-miR-125	Downregulated	Upregulated	Plasma	[37,111,119]
has-miR-29c	Upregulated	Downregulated	Plasma	[111,123]
has-miR-191	Upregulated	Upregulated	Plasma	[111,124,125]

## Abbreviations

The following abbreviations are used in this manuscript:

Abs	Apoptotic bodies
Ago	Argonautes
ARE	AU-rich element
AR	Androgen receptor
BC	Breast cancer
Bcl2	B-cell leukemia/lymphoma 2 protein
C. elegans	Caenorhabditis elegans
CSC	Cancer stem cell
CRISPR	Clustered regularly interspaced short palindromic repeats
cfc-miRs	Cell-free circulating miRNAs
c-miRs	Circulating miRNAs
ctDNA	Circulating tumor DNA
DBR1	Debranching enzyme 1
DGCR8	Di-George syndrome critical region 8
DICER	Endoribonuclease Dicer
DROSHA	Double-stranded RNA-specific endoribonuclease, type III
ddPCR	Droplet digital PCR
EBP1	ErbB3-binding protein 1
EC-miRs	Extracellular miRNAs
ECL	Electrochemiluminescence
EMT	Epithelial–mesenchymal transition
E2	estradiol
ER	Estrogen receptor
eRNA	Enhancer RNAs
ERα	Estrogen receptor alpha
e-siRNA	Endogenous small interfering RNAs
FXR1	Fragile X mental retardation-related protein 1
GPR30	G protein-coupled estrogen receptor 1
HDL	High-density lipoprotein
HER2	Human epidermal growth factor receptor-2
HER2+	Human epidermal growth factor receptor-2 positive
HRs	Hormone receptors
HRs	Hormone receptors
Lum A	Luminal A
LFAs	Lateral flow assays
Lum B	Luminal B
LB	Liquid Biopsy
lncRNAs	Long-noncoding RNAs
MEK/MAPK	Mitogen-activated protein kinase kinase
MS	Mass spectrometry
MBC	Metastatic breast cancer
MiRNAs	Micro ribonucleic acids
mRNA	messenger ribonucleic acid
MVBs	Multi-vesicular body
MYC	Myelocytomatosis oncogene
NAC	Neoadjuvant chemotherapy
NDD	Neurodevelopmental diseases
Nt	Nucleotides
P4	Progesterone
P-bodies	Processing bodies
pM	Pico Molar
pCR	Pathological complete response
PDCD	Programmed cell death
PDCD4	Programmed cell death protein 4
piRNAs	Piwi-interacting RNAs
Pol II	Polymerase II
PR	Progesterone receptor
pri-miRNA	Primary miRNA
PTEN	Phosphatase and TENsin homolog
Ran-GTPase	RAS-related nuclear protein-guanosine-5′-triphosphatase
RISC	RNA-induced silencing complex
RNAa	RNA activation
RNP	Ribonucleoprotein
SERS	Surface-enhanced Raman spectroscopy
snoRNA	Small nucleolar RNA
SOCS1	Suppressor of cytokine signaling 1
ssRNAs	Single-stranded RNAs
TDMD	Target-directed miRNA degradation
TME	Tumor microenvironment
TNBC	Triple-negative breast cancer
TP53INP1	Tumor protein 53-induced nuclear protein 1
tRNAs	Transfer RNAs
Tsp	Tumor suppressor
TWIST1	Twist-related protein 1
UTR	Untranslated region
XPO-5	Exportin-5
ZEB1	Zinc finger E-Box binding homeobox 1
ZEB2	Zinc finger E-Box binding homeobox 2
